# ESI FTICR-MS Analysis of Larvae from the Marine Sponge *Luffariella variabilis*

**DOI:** 10.3390/md8010190

**Published:** 2010-01-22

**Authors:** Cherie A. Motti, Piers Ettinger-Epstein, Richard H. Willis, Dianne M. Tapiolas

**Affiliations:** 1 Australian Institute of Marine Science, Townsville, Queensland, 4810, Australia; E-Mails: r.willis@aims.gov.au (R.H.W.); d.tapiolas@aims.gov.au (D.M.T.); 2 AIMS@JCU, Sir George Fisher Building, James Cook University, Townsville, 4811, Queensland, Australia; E-Mail: piers_ettinger_epstein@hotmail.com (P.E.-E.); 3 School of Marine and Tropical Biology, James Cook University, Townsville, 4811, Queensland, Australia

**Keywords:** Luffariella variabilis, manoalide monoacetate, larvae, ESI FTICR-MS, constitutive defence

## Abstract

The viviparous Great Barrier Reef sponge *Luffariella variabilis* (Poléjaeff 1884) contains a range of secondary metabolites, including manoalide (**1**) and manoalide monoacetate (**3**). ESI (+) FTICR-MS accurate mass determination has, for the first time, been used to detected the presence of **3** only in an organic extract of a single *L. variabilis* larva showing that the parentally produced **3** is sequestered in the larva. As **3** has previously been shown to have antibacterial and quorum sensing inhibition activity, and readily converts to **1**, which also exhibits similar activity, it may provide a chemical defence against predation and microbial attack.

## 1. Introduction

Most marine benthic invertebrates have complex life cycles characterised by sessile and planktonic phases [1]. Adults of sessile marine invertebrates that grow on exposed substrates and lack physical defences against predators are commonly defended by secondary metabolites [2–4]. Many of their early life forms, such as eggs and planktonic larvae, also rely on chemistry as a defensive mechanism to ensure their survival after release, dispersal, and settlement and are central to patterns of distribution and community structure [5]. Defensive chemicals have been reported in asteroid eggs [6], ascidian larvae [7], polychaetes [8], bryozoan larvae [9] and the egg masses of a nudibranch [10] whilst very little information relating to sponge larvae is available [11].

*Luffariella variabilis* is a cryptic coral reef sponge, generally found in aggregations in areas of low illumination and in high abundance on the Great Barrier Reef (GBR). It is gonochoristic and viviparous, with larvae approximately 400 μm × 200 μm in size [12]. The larvae are phototactic, initially swimming upwards after release, during which time they are vulnerable to predation [5].

*L. variabilis* contains an array of secondary metabolites including manoalide (**1**) [13] and its analogues seco-manoalide (**2**) [14], manoalide monoacetate (**3**) [14], luffariellins A and B (**4** and **5**) [16], the luffariolides [17–19] and the neomanoalides [14]. Manoalide (**1**) is an analgesic, possesses potent anti-inflammatory activity, irreversibly inhibits human synovial fluid PLA2 [20] as well as bee [21] and cobra venom [22] PLA2 and inhibits ornithine decarboxylase [23]. These activities have led to the use of manoalide in the prevention of post-surgical adhesion of tissues [24] and as a molecular tool in the study of psoriasis and skin cancers [23].

Manoalide (**1**) is commercially available although supply is still primarily by wild harvest. In a previous study, we reported that manoalide monoacetate (**3**, 35 to 70 mg g^−1^ dry weight of sponge) and manoalide (**1**, 15 to 20 mg g^−1^ dry weight) were consistently the most abundant compounds present in *L. variabilis* from the GBR making it an ideal target for either wild harvest or aquaculture for supply of **1** [25]. Seco-manoalide (**2**), luffariellin A (**4**), luffariellin B (**5**), and their acetoxy derivatives **6**, **7** and **8** [26] and were found to be 10 to 70 times less abundant and varied between 0 and 3 mg g^−1^ dry weight.

The ecological role of the manoalide class of compounds in the sponge is unknown. Duffy and Paul [27] reported that **1** significantly deterred feeding by fish in the field at natural concentrations of 0.5 and 1.5% of dry mass in low-quality food. Antimicrobial activity has also been reported for **1**–**3** [28] and more recently they were determined to be strong quorum sensing (QS) inhibitors [29].

Given that the *L. variabilis* larvae are vulnerable after initial release, the presence of a feeding deterrent/QS inhibitor/antimicrobial component in the larvae would seem beneficial. The fact that the sponge contains such chemistry raises the question; does *L. variabilis* sequester manoalide or related chemistry within its larvae? Our investigation was aimed at the detection of this class of secondary metabolites in the *L. variabilis* larvae using electrospray ionization Fourier Transform ion cyclotron resonance mass spectrometry (ESI FTICR-MS), a powerful mass spectrometric method capable of obtaining accurate exact mass measurements for elemental formula determination and ultra-high resolution over a large mass range (up to 70 kDa) [30].

## 2. Results and Discussion

ESI FTICR-MS analyses of manoalide (**1**) and manoalide monoacetate (**3**) were conducted in both positive and negative mode (Table 1). The [M + Na]^+^ ion was observed for both **1** and **3**. In the case of **1**, the hydrated, sodiated monomer [M + H_2_O + Na]^+^ and the hydrated, sodiated dimer [2M + 2H_2_O + Na]^+^ were also observed. For **3**, the sodiated dimer [2M + Na]^+^ and sodiated trimer [3M + Na]^+^ were detected.

Negative mode analysis of **1** resulted in the detection of the [M − H]^−^ ion, as expected, whereas analysis of **3** resulted in the cleavage of the acetyl group and dehydration to give an *m/z* corresponding to **1** [M − H_2_O − C_2_H_3_O − H]^−^. Altering the ionization conditions did not produce an [M − H]^−^ ion corresponding to **3**. Using negative mode conditions **1** and **3** could not be distinguished; all remaining analyses were conducted in positive mode.

**Figure 2:**
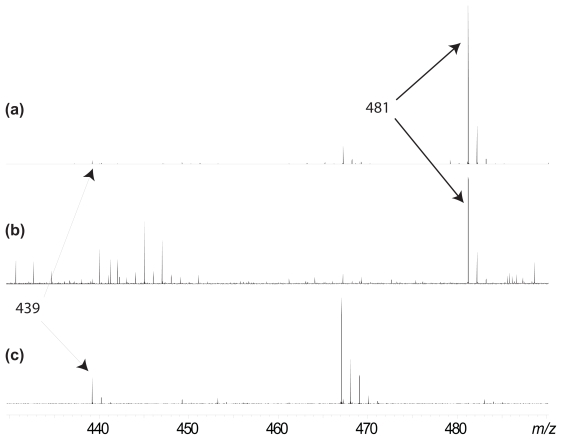
Portion of the molecular ion spectrum of: (a) Frozen, lyophilised sponge containing manoalide monoacetate (**3**, *m/z* 481) and trace amounts of manoalide (**1**, *m/z* 439), (b) frozen, lyophilised bulk larvae containing **3** only, and (c) thawed bulk larvae showing the presence of **1** and a total loss of **3**.

A gravid *L. variabilis* sponge, collected from Orpheus Island, GBR, was previously determined by HPLC and NMR to contain **3** in high abundance [25]. ESI (+) FTICR-MS of the crude dichloromethane (DCM, Burdick and Jackson HPLC grade) extract of the frozen, lyophilised sponge resulted in a series of signals between *m/z* 150 – 2,000, the molecular ion profile (Figure 2a), and in particular the presence of an intense sodiated ion ([M + Na]^+^, *m/z* 481.2521, Δ = 8 ppm) corresponding to **3** and a very weak ion ([M + H_2_O + Na]^+^, *m/z* 439.2449, Δ = 1 ppm) corresponding to **1**. Previous work on *L. variabilis* from the GBR [25] demonstrated that the production of **3** is hardwired, and that this compound is present in higher yields than other known analogues with the same molecular formula, including 25-acetoxyluffariellin A (**6**), 25-acetoxyluffariellin B (**7**) and 25-acetoxysecomanoalide (**8**), confirming the ion at *m/z* 481 was **3**.

Individual and bulk larvae were collected and immediately frozen [25]. Analysis of the crude DCM extract of the frozen, lyophilised bulk larvae detected the presence of an ion at *m/z* 481.2557, Δ = 1 ppm, corresponding to the sodiated ion of **3**, confirming the presence of **3** in the offspring (Figure 2b). No ions corresponding to **1** were observed. However, if the frozen sponge and larvae samples were allowed to thaw prior to extraction the *m/z* 481 ion was not detected. Instead, in both instances, an ion at *m/z* 439 was observed corresponding to the sodiated, hydrated ion of **1** (Figure 2).

**Figure 3:**
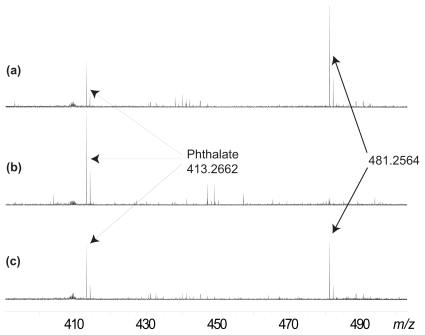
(a) Initial injection of DCM extract of an individual frozen larva, (b) injection of DCM:MeOH (1:1, v:v) blank and (c) Repeated injection of DCM extract of an individual frozen larva.

Repeating the analysis with a single larva resulted in the detection of an ion at *m/z* 481.2564, again within 1 ppm of expected 481.2561 for C_27_H_38_O_6_Na^+^, corresponding to **3**. To ensure there was no carry over between injections the syringe and spray needle were flushed three times with neat methanol (MeOH, *Omnisolv* HPLC grade) and then data acquired on a DCM-MeOH (1:1) blank between each sample. A second, repeated injection of the DCM extract from a single larva confirmed the presence of the peak at *m/z* 481. The presence of a persistent dioctyl phthalate ion at *m/z* 413.2662 [C_24_H_38_O_4_Na^+^], a plasticizer used extensively throughout society and now found throughout the environment, fortuitously provided an internal standard by which the spectra could be normalized for direct comparison (Figure 3). All 10 individual larva analysed were found to contain an ion corresponding to **3**, the same as that observed for the frozen sponge itself (Figure 3).

Lindquist and Hay [11] showed that diverse taxa of larvae are readily consumed by fishes and those that were unpalatable were found to be mostly brooded, highly coloured larvae that were released during the day, as are the *L. variabilis* larvae. Fine-scale temporal production of **3** in the sponge was shown to decrease over November and December, coinciding with the release of the brooded larvae [25]. The presence of **3** in these larvae strongly indicates that sequestration via parental transfer is occurring. Defensive properties of metabolites may also be altered temporally, typically following damage, via a very rapid process of chemical transformation known as activated defence. Mechanical tissue damage [31] and naturally occurring disease [32] of the sponge *Aplysina aerophoba* have been shown to induce an enzymatic bioconversion of isoxazoline alkaloids into aeroplysinin-1 and dienone. In contrast, wounding studies on *L. variabilis* [25] did not result in a change in the amounts of **3** indicating that the defence mechanism is more likely to be constitutive rather than activated; that is, it does not provide immediate protection but rather prepares the organism for future attacks. Interestingly, the acetylated compounds **3**, **6**, **7** and **8** have been shown to be labile in *L. variabilis* sponge tissue when samples were allowed to thaw prior to extraction, but were stable once isolated [26] suggesting that they are possibly being enzymatically transformed and/or degraded into their hydroxylated counterparts. The presence of the acetylated compound **3** in the larvae may provide a constitutive defence against predation, possibly by enzymatic mediated hydrolysis into **1**, a fish antifeedant, and thus might be of ecological importance for the survival of the larval stage.

Microorganisms are ubiquitous in the marine environment. Benthic invertebrates and their planktonic larvae could be expected to be susceptible to microbial attack. Many pathogenic bacteria rely on QS, a chemically driven mechanism employed to monitor density-dependent cell–cell communication and gene regulation, enabling the bacteria to control the change in behaviour from single-cell organisms to that of a colony and to co-ordinate their virulence. Both **1** and **3** have been shown to have antimicrobial activity and moreover to be strong QS inhibitors [29]. The presence of **3** and its possible enzymatic conversion to **1** may provide *L. variablis* larvae with a chemical means of resisting colonisation by potential pathogens as well as regulating microbial populations [33].

Schmitt *et al*. have shown that bacterial communities associated with larvae from the viviparous demosponge *Ircinia felix* are vertically transmitted from the parent and is an important mechanism for the establishment of the sponge-microbe association [34]. To date very little is understood regarding the microbial populations associated with *L. variabilis*, which may comprise up to 60% of sponge biomass, or indeed whether the sponge itself or the sponge-associated microbes are responsible for the production of the manoalide class of compounds. Given this, the possibility that microbial symbionts are producing the compound within the larvae cannot be discounted.

Component identification within crude extracts at sub nanogram scale can be achieved in a single ESI FTICR-MS experiment without separation and in a fraction of the time usually required for the traditional bioassay-guided fractionation approach to chemical ecology whilst minimizing possible adverse ecological effects. The application of ESI FTICR-MS techniques described here has allowed, for the first time, molecular formula information about individual constituents, namely **1** and **3**, to be derived from a crude extract of a sponge, its larvae and indeed of an individual larva with minimal preparation and no fractionation. The high-resolution and routine accurate mass capability of the ESI FTICR-MS technique adds another dimension to the arsenal available to investigate the chemistry of the sponge and its larvae at ecologically relevant concentrations.

## 4. Experimental Section

### 4.1. Animal material

A gravid *L. variabilis* sponge (order Dictyoceratida, family Thorectidae) was collected on a shallow coral reef slope (4–8 m) by Scuba at Orpheus Island in the Palm Islands group (18° 35′ 37″ S 146° 29′ 07″ E), GBR, Queensland, Australia, on 16 November 2005, AIMS voucher specimen number 27405. The sponge, collected based on its chemical profile determined from previous field sampling [25], was identified as gravid by removing a ~1 cm^3^ piece of mesohyl and placing it into a gonad fixative consisting of formalin, acetic acid and calcium chloride, within 1 h of collection [25]. Samples were fixed for 24 h before visually checking for the presence of larvae. The sponge was placed in a separate flow through aquarium at AIMS and acclimatised for 1 month. Sponge material was collected under AIMS permit G05/11866.1 and JCU permit G03/8695.1.

### 4.2. Larval collection

Larvae displayed phototactic behaviours swimming upwards upon release for brief periods (40 min) enabling collection in an inverted container. Individual larva were placed in a deep well plate and immediately frozen. A mesh trap was then placed over the gravid sponge to collect bulk released larvae, which were pooled and immediately frozen. All larvae were stored at −20 °C.

### 4.3. Standards

Manoalide (**1**) and manoalide monoacetate (**3**), previously isolated from frozen, lyophilised *L. variabilis* and purified as standards [25], were prepared in DCM-MeOH (1:1) at a final concentration of 200 μg mL^−1^.

### 4.4. Sample preparation

Sponge sample: a 1 cm^3^ piece of frozen sponge was lyophilised prior to extraction in 2 mL DCM and sonicated for 5 mins. A second 1 cm^3^ frozen piece of sponge was thawed prior to extraction in 2 mL DCM and sonicated for 5 mins. A 10 μL aliquot of each extract was diluted into 500 μL MeOH.

Bulk larvae samples: the bulk frozen larvae sample (n = 198) was divided into two. The first half was lyophilised and extracted directly in 500 μL DCM to which 500 μL MeOH was added and sonicated for 30 s. The second half was allowed to thaw completely before extraction in 500 μL DCM followed by addition of 500 μL MeOH and sonication for 30 s.

Individual larval samples: ten individual frozen larvae from the sponge were lyophilised then extracted separately in 200 μL DCM and sonicated for 30 s followed by the addition of 200 μL MeOH. Every alternate analysis was a blank of 1 mL DCM-MeOH (1:1) to ensure there was no carry over from one sample to the next.

All samples were analysed by direct injection into the ESI source of the FTICR-MS within 60 s of preparation.

### 4.5. ESI FTICR-MS

ESI FTICR-MS measurements were performed on an unmodified Bruker BioAPEX 47e mass spectrometer equipped with an Analytica of Branford model 103426 (Branford, CT, USA) electrospray ionisation (ESI) source in both positive and negative mode. Direct infusion of the sample was carried out using a Cole Palmer 74900 syringe pump at a rate of 100 μL h^−1^. The instrument was externally calibrated using a 0.1 mg mL^−1^ methanolic solution of CF_3_COONa (Sigma Aldrich) over *m/z* 150–2,000. For detailed information on the instrumentation please refer to Supporting Information accompanying Motti *et al*. [35].

## Figures and Tables

**Figure 1:**
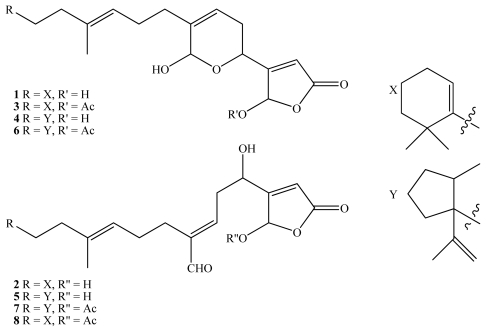
Stuctures of manoalide (**1**), secomanoalide (**2**), manoalide monoacetate (**3**), lufferiellin A (**4**), lufferiellin B (**5**), 25-acetoxyluffariellin A (**6**), 25-acetoxyluffariellin B (**7**) and 25-acetoxyseco-manoalide (**8**).

**Table 1:** Ions observed for manoalide (**1**) and manoalide monoacetate (**3**) in positive and negative mode ESI FTICR-MS.

No.	mode	Formula	Corresponding ion	*m/z* calculated	*m/z* observed	Δ ppm
**1**	+	C_25_H_35_O_4_^+^	[M + H]^+^	399.2530	not obs	-
		C_25_H_34_O_4_Na^+^	[M + Na]^+^	421.2349	421.2343	1.4
		C_25_H_36_O_5_Na^+^	[M + H_2_O + Na]^+^	439.2455	439.2448	1.6
		C_50_H_70_O_10_Na^+^	[2M + 2H_2_O + Na]^+^	855.5018	855.5015	0.4

**1**	−	C_25_H_33_O_4_^−^	[M − H]^−^	397.2384	397.2388	1.0
		C_50_H_71_O_10_^−^	[2M + 2H_2_O − H]^−^	831.5053	831.5055	0.2

**3**	+	C_27_H_39_O_6_^+^	[M + H]^+^	459.2741	not obs	-
		C_27_H_38_O_6_Na^+^	[M + Na]^+^	481.2561	481.2567	1.2
		C_54_H_76_O_12_Na^+^	[2M + Na]^+^	939.5229	939.5216	1.4
		C_81_H_114_O_18_Na^+^	[3M + Na]^+^	1397.7897	1397.7821	5.4

**3**	−	C_27_H_37_O_6_^−^	[M − H]^−^	457.2596	not obs	-
		C_25_H_33_O_4_^−^	[M − H_2_O − C_2_H_3_O]^−^	397.2384	397.2384	0
